# Inactivated polio vaccines from three different manufacturers have equivalent safety and immunogenicity when given as 1 or 2 additional doses after bivalent OPV: Results from a randomized controlled trial in Latin America

**DOI:** 10.1016/j.vaccine.2017.04.041

**Published:** 2017-06-16

**Authors:** Eduardo Lopez-Medina, Mario Melgar, James T. Gaensbauer, Ananda S. Bandyopadhyay, Bhavesh R. Borate, William C. Weldon, Ricardo Rüttimann, Joel Ward, Ralf Clemens, Edwin J. Asturias

**Affiliations:** a Department of Pediatrics, Universidad del Valle and Centro de Estudios en Infectología Pediátrica, Cali, Colombia; b Hospital Roosevelt and University Francisco Marroquin School of Medicine, Guatemala City, Guatemala; c Department of Pediatrics, University of Colorado School of Medicine, Aurora, CO, USA; d Center for Global Health and Department of Epidemiology, Colorado School of Public Health, Aurora, CO, USA; e Bill & Melinda Gates Foundation, Seattle, WA, USA; f Fred Hutchinson Cancer Research Center, Seattle, WA, USA; g Centers for Disease Control and Prevention, Atlanta, GA, USA; h Fighting Infectious Diseases in Emerging Countries (FIDEC), Miami, FL, USA; i Department of Pediatrics, Harbor-UCLA Medical Center, Geffin School of Medicine, University of California at Los Angeles, CA, USA; j Global Research in Infectious Diseases (GRID), Rio de Janeiro, Brazil

**Keywords:** IPV, inactivated poliovirus vaccine (trivalent, serotypes 1, 2 & 3), mOPV2, monovalent oral poliovirus vaccine (serotype 2), bOPV, bivalent oral poliovirus vaccine (serotypes 1 & 3), tOPV, trivalent oral poliovirus vaccine (serotypes 1, 2 & 3), DTPw-HBV-Hib, diphtheria-tetanus-whole cell pertussis-hepatitis B-*Haemophilus influenzae* type b combination vaccine, Poliovirus, Vaccination, Humoral immunity, Intestinal immunity

## Abstract

**Background:**

Since April 2016 inactivated poliovirus vaccine (IPV) has been the only routine source of polio type 2 protection worldwide. With IPV supply constraints, data on comparability of immunogenicity and safety will be important to optimally utilize available supplies from different manufacturers.

**Methods:**

In this multicenter phase IV study, 900 Latin American infants randomly assigned to six study groups received three doses of bOPV at 6, 10 and 14 weeks and either one IPV dose at 14 weeks (groups SP-1, GSK-1 and BBio-1) or two IPV doses at 14 and 36 weeks (groups SP-2, GSK-2 and BBio-2) from three different manufacturers. Children were challenged with mOPV2 at either 18 (one IPV dose) or 40 weeks (two IPV doses) and stools were collected weekly for 4 weeks to assess viral shedding. Serum neutralizing antibodies were measured at various time points pre and post vaccination. Serious adverse events and important medical events (SAE and IME) were monitored for 6 months after last study vaccine.

**Results:**

At week 18, 4 weeks after one dose of IPV, overall type 2 seroconversion rates were 80.4%, 80.4% and 73.3% for SP-1, GSK-1 and BBio-1 groups, respectively; and 92.6%, 96.8% and 88.0% in those who were seronegative before IPV administration. At 40 weeks, 4 weeks after a second IPV dose, type 2 seroconversion rates were ≥99% for any of the three manufacturers. There were no significant differences in fecal shedding index endpoint (SIE) after one or two IPV doses (SP: 2.3 [95% CI: 2.1–2.6]); GSK: 2.2 [1.7–2.5]; BBio 1.8 [1.5–2.3]. All vaccines appeared safe, with no vaccine-related SAE or IME.

**Conclusion:**

Current WHO prequalified IPV vaccines are safe and induce similar humoral and intestinal immunity after one or two doses.

The parent study was registered with ClinicalTrials.gov, number NCT01831050.

## Highlights

• Since April 2016 type 2 polio immunization is only possible with inactivated polio vaccines (IPV).
• Global IPV supply is currently under pressure, being supplied by only four manufacturers.
• We previously confirmed the effectiveness of mixed bOPV and IPV schedules from one manufacturer.
• We compared intestinal and humoral immunity of IPV from 3 WHO prequalified manufacturers.
• All three WHO prequalified IPV were similarly immunogenic when 1 or 2 doses were given with bOPV.

## 1 Introduction

Since April 2016, all countries using OPV have switched to bivalent OPV (bOPV) as part of the final steps for global eradication of all-cause poliomyelitis. bOPV maintains protection against type 1 and 3 polioviruses, but leaves young children vulnerable to infection by type 2 vaccine-derived polioviruses [1], [2]. To strengthen population immunity and ensure all children are protected against type 2 polioviruses in countries that are polio-endemic or at high risk of importation of the virus, the WHO Strategic Advisory Group of Experts (SAGE) recommends at least one dose of IPV, given with the third dose of bOPV at 14 weeks of age or older to minimize interference from maternally-derived antibodies [3]. In countries with 90–95% immunization coverage and a low importation risk, IPV-OPV sequential schedules can be used to minimize the risk of vaccine-associated paralytic polio (VAPP) [4].

Universal IPV use will now become routine to induce adequate type 2 protection, and to boost individual and population type 1 and 3 immunity in the final stages of global polio eradication. As wild-type polio is eradicated, IPV will become the only polio vaccine to sustain population immunity and prevent reemergence. However, currently only four WHO-prequalified manufacturers (Sanofi Pasteur, GlaxoSmithKline, Bilthoven Biologicals, and the Staten Serum Institute) supply wild-type IPV. Manufacturing capacity is limited by challenges to scaling up bulk production and establishing additional production sites given the Global Action Plan III regulations, putting a major strain on global IPV supply [5].

Although all four WHO-prequalified IPV vaccines are well established, no direct evidence exists on their comparative immunogenicity and safety. In our previously reported study on the immunity induced by bOPV-IPV schedules (6) a secondary objective was to compare the safety, humoral and intestinal immunity between three of these different IPVs.

## 2 Methods

This phase IV open-label, observer-blind, multicenter, randomized, controlled study was conducted from May 2013 to February 2015 at six sites in Colombia, the Dominican Republic, Guatemala and Panama. The protocol was approved by all local Ethics Committees and by National Regulatory Authorities, the Colorado Multiple Institutional and the Western Institutional Review Boards. Parents/guardians provided written informed consent before enrolment. An independent Data Safety Monitoring Board (DSMB) monitored benefit-risk throughout the study.

We previously reported the primary objective, to assess superiority of bOPV-IPV schedules over bOPV-only, based on humoral and intestinal serotype 2 immunity (fecal shedding post-challenge) [6], and now report the secondary objective to compare type 2 poliovirus immunogenicity and safety of three IPV vaccines.

### 2.1 Participants

Eligible subjects were healthy, 6 week-old, full-term infants, attending well baby clinics for their first polio vaccinations. Inclusion, exclusion and withdrawal criteria were described previously [6], the major inclusion criteria being no prior polio vaccination history and no siblings/household members who had recently received or were scheduled to receive OPV.

### 2.2 Vaccines and schedules

Three IPVs (Supplementary Table 1) from Sanofi Pasteur (SP; Marcy L’Etoile, France), GlaxoSmithKline (GSK; Wavre, Belgium), and Bilthoven Biologicals BV (BBio; Bilthoven, the Netherlands) were studied. In the parent study enrolled infants were randomly allocated by permuted block randomization (using a computer-generated list, block size 36) to nine groups, six of which we describe here for comparison of different IPV responses. All infants received three bOPV doses, at 6, 10, 14 weeks, plus one manufacturer’s IPV at 14 weeks (groups SP-1, GSK-1 and BBio-1), or two IPV doses at 14 and 36 weeks (groups SP-2, GSK-2 and BBio-2). Children were challenged with mOPV2 at either 18 or 40 weeks of age, four weeks after their last IPV dose. SP-1 and SP-2 groups correspond to Groups 4 and 5 in the previously published results [6]. Concomitantly at 6, 10, 14 weeks, DTwP-HBV-Hib (Quinvaxem™, Novartis Vaccines, Marburg, Germany), pneumococcal conjugate vaccine (Synflorix™, GlaxoSmithKline, Rixensart, Belgium or Prevnar™, Pfizer, New York, USA), and rotavirus vaccine (Rotarix™, GlaxoSmithKline, Rixensart, Belgium) were administered according to each country’s routine immunization requirements. IPV was injected intramuscularly in the left thigh, other intramuscular vaccines were given in the right thigh. Blinding to which IPV was maintained at administration and in all subsequent laboratory analyses. Additional IPV doses were administered after the last study sample collection to ensure all children received at least two doses of IPV by study end.

### 2.3 Humoral immunogenicity

Blood (1.5–3 mL) was drawn in all groups at 6 and 14 weeks, and subsequently at 18 and 19 weeks (immediately prior and one week after mOPV2 challenge to assess priming) in the one-dose IPV groups, or at 36 and 40 weeks in the two-dose IPV groups. Serum neutralizing antibodies, measured at the Polio and Picornavirus Laboratory, Centers for Disease Control and Prevention, Atlanta, USA, were expressed as median log_2_ titers, seropositivity rates (group proportions with titer ≥8), and seroconversion rates, as described previously [6]. The overall study seroconversion rate was defined as the proportion of seronegative infants who became seropositive, and of pre-vaccination seropositive infants having titers ≥4-fold higher than expected levels of maternally-derived antibody, assuming an exponential decay with a half-life of 24 days [7]. The specific IPV seroconversion rate was defined as a 4-fold or greater rise among seroprotected or titers ≥8 among 14-week seronegative, at 4, 22 or 26 weeks after administration of one or two IPV depending on the schedule.

### 2.4 Intestinal immunity

Stool samples (5–10 mg) collected before and at weekly intervals over four weeks after mOPV2 challenge were analyzed for type 2 viral titers as described previously (6). Numbers and proportions of shedders, infants who had a log_10_ titer of 2.75 or higher, were calculated each week post-challenge. Intestinal immunity was assessed using a composite shedding index endpoint (SIE), computed as the mean of stool log_10_ viral titers at Days 7, 14, 21 and 28 post-challenge, assigning zero values to samples not positive for type 2 by RT-PCR.

### 2.5 Safety assessment

As the study used licensed and WHO prequalified vaccines, safety assessment was limited to recording serious adverse events (SAE) and important medical events (IME) throughout the study until 6 months after the last vaccine dose. SAE were defined as death or events that caused persistent or significant disabilities, or that required hospitalization, and IME as medically significant events that were not SAEs but required medical intervention or consultation.

### 2.6 Sample size

Sample sizes calculated for primary objectives of the parent study have been described previously (6). Sample sizes for GSK-2 and BBio-2 were selected to enable comparison to the corresponding bOPV-only regimen, assuming maximum seroconversion rates of 50% for type 2 in the bOPV-only group, and 90% for all regimens for all remaining serotypes. For evaluable group sizes of 152 (planned enrollment of 190, assuming 20% dropout rate), there was power of 0.80–0.86 to declare joint non-inferiority for all serotypes with margin 10%, with an overall type I error rate of 5%. Group sizes of 50 were selected for secondary comparisons of manufacturers among the 1-dose boosted IPV regimens to allow moderate precision for estimated rates of seroconversion and seroprotection. Assuming seroconversion rates of 90% for bOPV + 1IPV and 98% for bOPV + 2IPV, power for the equivalence comparisons considered here ranges from 12% (GSK-1 vs BBio-1) to 41% (SP-1 vs GSK-1/BBio-1) to >99% (SP-2 vs GSK-2 vs BBio-2).

### 2.7 Statistical analysis

The per-protocol (PP) cohort, consisting of all study participants who received all immunization(s) scheduled for the allocated group with samples available for analysis, was used for this secondary analysis. Results from binary outcomes (seroconversion, seropositivity) were summarized using rates with Wilson confidence intervals. Confidence intervals for differences between rates were computed using asymptotic normal methods. Medians with 95% confidence intervals were computed for continuous outcomes such as neutralization titers and shedding, using the bootstrap method for confidence intervals, and with reverse cumulative distribution curves. Geometric mean titers (GMT) were also used to compare effects of IPV administration in those seropositive vs. seronegative at different time-points. Equivalence in rates of seroconversion to each serotype was tested for immunogenicity responses after 1 and 2 doses from different IPV manufacturers [8]. A 10% margin was used for primary endpoint binary comparisons, which was re-used here as a two-sided margin for equivalence comparisons among manufacturers, each at level α = 0.05. Overall equivalence of one manufacturer to another was claimed if an equivalence outcome was obtained for each of the 3 endpoints (seroconversion, neutralization titers and SIE). Equivalence margins for these 3 endpoints were set at ±2/3 log_2_ for neutralization titers, ±1.0 log_10_, ±1.0 log_10_ for SIE. Neutralization titers, estimated by the Spearman-Kärber method [9] were calculated as the reciprocal of the calculated 50% estimate, with minimum and maximum values of 2.5 and 10.5 log_2_ titer, respectively. No corrections for multiple comparisons were made.

## 3 Results

### 3.1 Study population

**Fig. 1:**
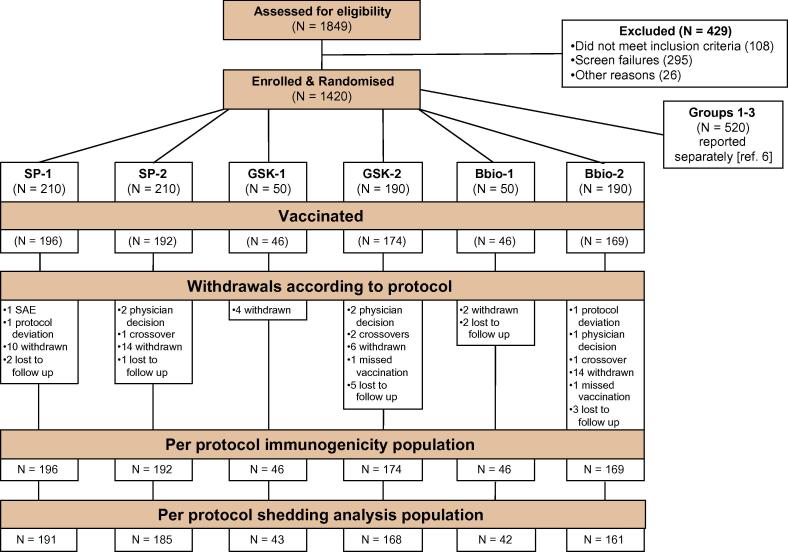
Flow chart of the six study groups showing attrition to the Per Protocol populations for immunogenicity and shedding analyses.

Of 900 infants randomly assigned to the six study groups included for the comparison between different IPVs, all received at least one study vaccine and are included in the safety analysis; 823 (91.4%) received all vaccinations according to protocol and provided blood samples, and 790 (87.8%) provided stool for the shedding analyses (Fig. 1). Drop-outs were mainly due to parental withdrawal (n = 50), loss to follow-up (n = 13), protocol violations including receipt of the wrong vaccine (n = 6), and exclusions by the local investigator (n = 5). Baseline demographics were comparable across the study groups (Table 1) and between countries (not shown). There were no statistically significant differences in neutralizing antibody titers at study entry at 6 weeks for any serotype across study groups (Supplementary Table 2). For type 2, baseline seroconversion rates prior to IPV at week 14 were 8.8% for SP-1, 6.5% for GSK-1, 10.9% for BBio-1 (p = 0.74), and 8.3% for SP-2, 10.9% for GSK-2 and 10.7% for BBio-2 (p = 0.65)(Table 2).

**Table 1:** Polio vaccines administered in the six study groups, and demographic characteristics at first vaccination in each group.

IPV manufacturer		Sanofi Pasteur		GlaxoSmithKline Bio		Bilthoven Bio	
Group		SP-1^a^	SP-2^a^	GSK-1	GSK-2	BBio-1	BBio-2
Enrolled		210	210	50	190	50	190
Vaccinated with IPV		196	192	46	174	46	169
Polio vaccines at weeks indicated	bOPV	6, 10, 14	6, 10, 14	6, 10, 14	6, 10, 14	6, 10, 14	6, 10, 14
	1st IPV	14	14	14	14	14	14
	2nd IPV	None	36	None	36	None	36
	mOPV2	18	40	18	40	18	40
Weeks when Viral shedding assessed		18–22	40–44	18–22	40–44	18–22	40–44
Male, n%		118 60	89 46	24 52	95 55	27 59	83 49
Mean age in days ±SD		45.1 ±5.8	44.9 ±6.3	43.7 ±6.1	44.4 ±5.9	43.0 ±6.2	45.1 ±6.2
Breastfeeding, n%		192 98	191 99	46 100	172 99	45 98	168 99
Day care, n%		6 3	6 3	1 2	9 5	0 0	5 3
Family size		4	5	5	5	4	4
(min, max)		(0, 16)	(2, 12)	(3, 10)	(2, 19)	(2, 16)	(2, 11)

a These two groups correspond to Groups 4 and 5 in the parent study [6].

**Table 2:** Seroconversion (95% CI) against the three polio serotypes in the six study groups who received one or two doses of IPV from the three different manufacturers (PP data-set).

One dose of IPV at week 14					Two doses of IPV at weeks 14 and 36				
Week	SP-1	GSK-1	BBio-1	Combined	Week	SP-2	GSK-2	BBio-2	Combined
*Serotype 1*									
**14**	*191/194*	*42/46*	*46/46*	*279/286*	**14**	*190/192*	*170/174*	*167/169*	*527/535*
	**98.5**	**91.3**	**100**	**97.6%**		**99.0**	**97.7**	**98.8**	**98.5%**
	(95.5–99.5)	(79.7–96.6)	(92.3–100)	(95.0–98.8)		(96.3–99.7)	(94.2–99.1)	(95.8–99.7)	(97.1–99.2)
**18**	*194/194*	*46/46*	*45/45*	*285/285*	**36**	*191/192*	*173/173*	*169/169*	*533/534*
	**100**	**100**	**100**	**100%**		**99.5**	**100**	**100**	**99.8%**
	(98.1–100)	(92.3–100)	(92.1–100)	(98.7–100)		(97.1–99.9)	(97.8–100)	(97.8–100)	(99.0–100)
**19**	*193/193*	*45/45*	*45/45*	*283/283*	**40**	*192/192*	*173/174*	*169/169*	*534/535*
	**100**	**100**	**100**	**100%**		**100**	**99.4**	**100**	**99.8%**
	(98.0–100)	(92.1–100)	(92.1–100)	(98.7–100)		(98.0–100)	(96.8–99.9)	(97.8–100)	(99.0–100)

***Serotype 2***									
**14**	*17/194*	*3/46*	*5/46*	*25/286*	**14**	*16/192*	*19/174*	*18/169*	*53/535*
	**8.8**	**6.5**	**10.9**	**8.7%**		**8.3**	**10.9**	**10.7**	**9.9%**
	(5.5–13.6)	(2.2–17.5)	(4.7–23.0)	(6.0–12.6)		(5.2–13.1)	(7.1–16.4)	(6.8–16.2)	(7.6–12.7)
**18**	*156/194*	*37/46*	*33/45*	*226/285*	**36**	*143/192*	*127/173*	*145/169*	*415/534*
	**80.4**	**80.4**	**73.3**	**79.3%**		**74.5**	**73.4**	**85.8**	**77.7%**
	(74.3–85.4)	(66.8–89.3)	(59.0–84.0)	(74.2–83.6)		(67.9–80.1)	(66.4–79.4)	(79.7–90.3)	(74.0–81.0)
**19**	*176/193*	*45/45*	*39/45*	*260/283*	**40**	*192/192*	*173/174*	*169/169*	*534/535*
	**91.2**	**100**	**86.7**	**91.9%**		**100**	**99.4**	**100**	**99.8%**
	(86.4–94.4)	(92.1–100)	(73.8–93.7)	(88.1–94.5)		(98.0–100)	(96.8–99.9)	(97.8–100)	(99.0–100)

***Serotype 3***									
**14**	*186/194*	*43/46*	*45/46*	*274/286*	**14**	*191/192*	*169/174*	*161/169*	*521/535*
	**95.9**	**93.5**	**97.8**	**95.8%**		**99.5**	**97.1**	**95.3**	**97.4%**
	(92.1–97.9)	(82.5–97.8)	(88.7–99.6)	(92.8–97.6)		(97.1–99.9)	(93.5–98.8)	(90.9–97.6)	(95.7–98.4)
**18**	*194/194*	*46/46*	*44/45*	*284/285*	**36**	*192/192*	*172/173*	*167/169*	*531/534*
	**100**	**100**	**97.8**	**99.6%**		**100**	**99.4**	**98.8**	**99.4%**
	(98.1–100)	(92.3–100)	(88.4–99.6)	(98.0–99.9)		(98.0–100)	(96.8–99.9)	(95.8–99.7)	(98.4–99.8)
**19**	*191/193*	*45/45*	*44/45*	*280/283*	**40**	*191/192*	*174/174*	*169/169*	*534/535*
	**99.0**	**100**	**97.8**	**98.9%**		**99.5**	**100**	**100**	**99.8%**
	(96.3–99.7)	(92.1–100)	(88.4–99.6)	(96.9–99.6)		(97.1–99.9)	(97.8–100)	(97.8–100)	(99.0–100)

### 3.2 Humoral immunity after one IPV dose

Of 285 infants assessed at 14 weeks, after 2 doses of bOPV but before a dose of IPV, overall seroconversion rates against types 1 and 3 were 97.6% and 95.8% across the three groups (Table 3). At 18 weeks, 4 weeks after the third bOPV and one dose of IPV, overall type 2 seroconversion reached 80.4%, 80.4% and 73.3% in the SP-1, GSK-1 and BBio-1 groups, respectively, not meeting our definition of equivalence between BBio-1 and SP-1 or GSK-1 (two-sided test p-values of 0.56). Moreover, when considering only those seronegative by 14 weeks of age, the type 2 seroconversion was 92.6% for SP-1, 96.8% for GSK-1 and 88.0% for BBio-1, respectively (Table 3). Amongst all infants (seropositive and seronegative at baseline) at 36 weeks (22 weeks after one dose of IPV), overall seroconversion rates in the SP-2, GSK-2 and BBio-2 groups were 74.5%, 73.4% and 85.8% respectively, again not meeting the equivalence definition between BBio vs. SP and BBio vs. GSK (p > 0.63) (Table 2). Among all one-dose IPV groups, the type 2 antibody GMT was <20.3 at baseline, decreasing to <9.7 by 14 weeks (data not shown). However, four weeks after one dose of SP, GSK or BBio IPV type 2 GMTs increased to 35.8, 44.3, and 35.3, respectively. At 36 weeks, 22 weeks after one IPV dose in the SP-2, GSK-2 and BBio-2 groups type 2 GMTs were 26.2, 28.6, and 36.5, respectively. Median log_2_ titers against poliovirus types 1 and 3 reached maximum detection levels (>10.5) by 14 weeks in all groups (Supplementary Table 2). For type 2 titers, equivalence was demonstrated between the SP-1 and BBio-1 groups at 18 weeks and the SP-2 and GSK-2 groups at 36 weeks (p < 0.03).

**Table 3:** Seroconversion (95% CI) against Serotype 2 in the six study groups who received one or two doses of IPV from the three different manufacturers according to seropositivity at 6 and 14 weeks (PP data-set).

One dose of IPV at week 14					Two doses of IPV at weeks 14 and 36				
Week	SP-1	GSK-1	BBio-1	Combined	Week	SP-2	GSK-2	BBio-2	Combined
*Seropositive at 6 weeks*									
**14**	8/109	1/25	1/28	10/162	**14**	13/99	11/107	10/88	34/294
	**7.3%**	**4.0%**	**3.6%**	**6.2%**		**13.1%**	**10.3%**	**11.4%**	**11.6%**
	(3.8–13.8)	(0.7–19.5)	(0.6–17.7)	(3.4–11.0)		(7.8–21.2)	(5.8–17.5)	(6.3–19.7)	(8.4–15.7)
**18**	77/109	16/25	17/28	110/162	**36**	74/99	73/106	70/88	217/293
	**70.6%**	**64.0%**	**60.7%**	**67.9%**		**74.7%**	**68.9%**	**79.5%**	**74.1%**
	(61.5–78.4)	(44.5–79.8)	(42.4–76.4)	(60.4–74.6)		(65.4–82.3)	(59.5–76.9)	(70.0–86.7)	(68.8–78.8)
**19**	93/109	24/24	22/28	139/161	**40**	99/99	107/107	88/88	294/294
	**85.3%**	**100%**	**78.6%**	**86.3%**		**100%**	**100%**	**100%**	**100%**
	(77.5–90.8)	(86.2–100)	(60.5–89.8)	(80.2–90.8)		(96.3–100)	(96.5–100)	(95.8–100)	(98.7–100)

*Seronegative at 6 weeks*									
**14**	9/85	2/21	4/18	15/124	**14**	3/93	8/67	8/81	19/241
	**10.6%**	**9.5%**	**22.2%**	**12.1%**		**3.2%**	**11.9%**	**9.9%**	**7.9%**
	(5.7–18.9)	(2.6–28.9)	(9.0–45.2)	(7.5–19.0)		(1.1–9.1)	(6.2–21.8)	(5.1–18.3)	(5.1–12.0)
**18**	79/85	21/21	16/17	116/123	**36**	69/93	54/67	75/81	198/241
	**92.9%**	**100%**	**94.1%**	**94.3%**		**74.2%**	**80.6%**	**92.6%**	**82.2%**
	(85.4–96.7)	(84.5–100)	(73.0–99.0)	(88.7–97.2)		(64.5–82.0)	(69.6–88.3)	(84.8–96.6)	(76.8–86.5)
**19**	83/84	21/21	17/17	121/122	**40**	93/93	66/67	81/81	240/241
	**98.8%**	**100%**	**100%**	**99.2%**		**100%**	**98.5%**	**100%**	**99.6%**
	(93.6–99.8)	(84.5–100)	(81.6–100)	(95.5–99.9)		(96.0–100)	(92.0–99.7)	(95.5–100)	(97.7–99.9)

*Seropositive at 14 weeks*									
**18**	12/58	3/15	3/20	18/93	**36**	6/53	5/53	11/58	22/164
	**20.7%**	**20.0%**	**15.0%**	**19.4%**		**11.3%**	**9.4%**	**19.0%**	**13.4%**
	(12.2–32.8)	(7.0–45.2)	(5.2–36.0)	(12.6–28.5)		(5.3–22.6)	(4.1–20.2)	(10.9–30.9)	(9.0–19.5)
**19**	31/57	9/14	14/19	54/90	**40**	50/53	47/53	48/58	145/164
	**54.4%**	**64.3%**	**73.7%**	**60.0%**		**94.3%**	**88.7%**	**82.8%**	**88.4%**
	(41.6–66.6)	(38.8–83.7)	(51.2–88.2)	(49.7–69.5)		(84.6–98.1)	(77.4–94.7)	(71.1–90.4)	(82.6–92.5)

*Seronegative at 14 week*									
**18**	126/136	30/31	22/25	178/192	**36**	106/139	96/120	104/111	306/370
	**92.6%**	**96.8%**	**88.0%**	**92.7%**		**76.3%**	**80.0%**	**93.7%**	**82.7%**
	(87.0–96.0)	(83.8–99.4)	(70.0–95.8)	(88.1–95.6)		(68.5–82.6)	(72.0–86.2)	(87.5–96.9)	(78.5–86.2)
**19**	132/134	31/31	25/26	188/191	**40**	139/139	121/121	111/111	371/371
	**98.5%**	**100%**	**96.2%**	**98.4%**		**100%**	**100%**	**100%**	**100%**
	(94.7–99.6)	(89.0–100)	(81.1–99.3)	(95.5–99.5)		(97.3–100)	(96.9–100)	(96.7–100)	(99.0–100)

In seronegative infants at 14 weeks, 14/192 (7.3%) did not seroconvert to type 2 by 18 weeks after one IPV dose, but 10/191 (71%) did so one week after challenge with mOPV2 (Table 3), illustrating priming. Conversely, 75 of the 93 (80.6%) seropositive at 14 weeks did not seroconvert after one IPV dose by 18 weeks, but 54 of 90 (60%) did so after mOPV2 challenge. These seroconversion rates were equivalent for the three manufacturer groups.

### 3.3 Humoral immunity after two IPV doses

Of 534 infants given IPV at 14 and 36 weeks, overall seroconversion rates at week 36 (22 weeks after the first dose but before the second) were 99.8% for type 1 and 99.4% for type 3 for the three different IPVs (Table 2). While 100% (n = 371) of those seronegative for type 2 at 14 weeks seroconverted after two IPV doses, 88.4% (145/164) of those seropositive seroconverted by week 40 (p < 0.001)(Table 3).

Overall 99.8% seroconversion was achieved against all serotypes at 40 weeks, one-month after a second IPV dose, irrespective of manufacturer, and all groups had maximum detection titers (>10.5) against all poliovirus serotypes after the second dose of IPV.

### 3.4 Intestinal immunity: impact on poliovirus type 2 excretion

Proportions of subjects shedding fecal type 2 virus from day 7 to day 28 decreased by 53.1%, 57.2% and 58.1% in the SP-1, GSK-1 and BBio-1 groups, respectively. Infants challenged with mOPV2 after one dose of IPV from any manufacturer had an overall viral shedding index endpoint (SIE) of 2.5 (95%CI; 2.3, 2.9) with a median SIE of 0.0 after day 21 (Table 4). The SIE was equivalent among manufacturers after dose 1.

**Table 4:** Proportions of infants shedding polio type 2 virus in stools, with median log10 poliovirus titers, after challenge with mOPV2, and the Shedding Index Endpoint for the six study groups given one or two doses of IPV from the different manufacturers.

One dose of IPV at week 14 (mOPV2 at week 18)					Two doses of IPV at weeks 14 and 36 (mOPV2 at week 40)				
Day	SP-1	GSK-1	BBio-1	Combined	Day	SP-2	GSK-2	BBio-2	Combined
*n/N and Percentage (95% CI) with fecal shedding of polio type 2 virus per group*									
7	*140/193*	*32/44*	*39/46*	*211/283*	7	*140/188*	*126/172*	*101/168*	*367/528*
	**72.5%**	**72.7%**	**84.8%**	**74.6%**		**74.5%**	**73.3%**	**60.1%**	**69.5%**
	(65.8–78.3)	(58.1–83.7)	(71.8–92.4)	(69.2–79.3)		(67.8–80.2)	(66.2–79.3)	(52.6–67.2)	(65.5–73.3)
14	*108/192*	*24/44*	*30/44*	*162/280*	14	*108/190*	*84/170*	*81/164*	*273/524*
	**56.2%**	**54.5%**	**68.2%**	**57.9%**		**56.8%**	**49.4%**	**49.4%**	**52.1%**
	(49.2–63.1)	(40.1–68.3)	(53.4–80.0)	(52.0–63.5)		(49.7–63.7)	(42.0–56.9)	(41.8–57.0)	(47.8–56.4)
21	*90/192*	*16/45*	*20/44*	*126/281*	21	*63/189*	*57/169*	*33/165*	*153/523*
	**46.9%**	**35.6%**	**45.5%**	**44.8%**		**33.3%**	**33.7%**	**20.0%**	**29.3%**
	(40.0–53.9)	(23.2–50.2)	(31.7–59.9)	(39.1–50.7)		(27.0–40.3)	(27.0–41.1)	(14.6–26.8)	(25.5–33.3)
28	*65/191*	*14/45*	*16/45*	*95/281*	28	*54/191*	*44/171*	*31/167*	*129/529*
	**34.0%**	**31.1%**	**35.6%**	**33.8%**		**28.3%**	**25.7%**	**18.6%**	**24.4%**
	(27.7–41.0)	(19.5–45.7)	(23.2–50.2)	(28.5–39.5)		(22.4–35.0)	(19.8–32.8)	(13.4–25.1)	(20.9–28.2)

*Median log10 poliovirus type 2 concentration in shedders*									
7	**4.6**	**4.6**	**4.8**	**4.6**	7	**5.4**	**5.2**	**4.6**	**5.1**
14	**2.9**	**2.8**	**3.2**	**2.9**	14	**2.8**	**0.0**	**0.0**	**2.8**
21	**0.0**	**0.0**	**0.0**	**0.0**	21	**0.0**	**0.0**	**0.0**	**0.0**
28	**0.0**	**0.0**	**0.0**	**0.0**	28	**0.0**	**0.0**	**0.0**	**0.0**

*Shedding Index Endpoint (SIE)*									
**SIE**	**2.6**	**2.2**	**2.5**	**2.5**	**SIE**	**2.3**	**2.2**	**1.8**	**2.2**
(95% CI)	(2.2–3.0)	(1.4–3.0)	(2.2–3.4)	(2.3–2.9)	(95% CI)	(2.1–2.6)	(1.7–2.5)	(1.5–2.2)	(1.8–2.3)
[n]	[191]	[43]	[42]	[276]	[n]	[185]	[168]	[161]	[514]

**Fig. 2:**
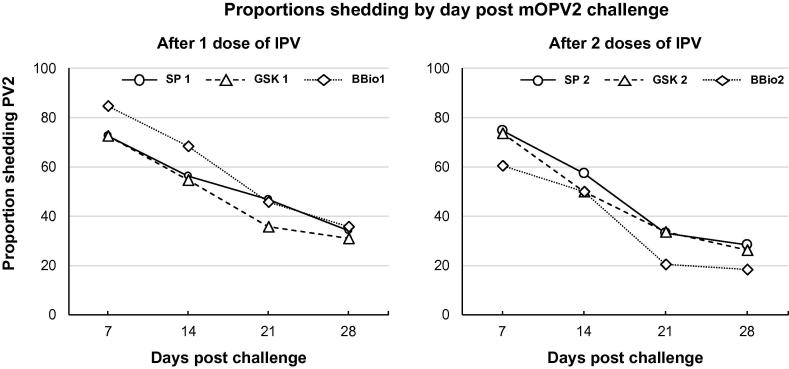
Rate of excretion (%) of poliovirus type 2 by IPV manufacturer and days post challenge with mOPV2.

After two IPV doses, proportions of shedders decreased between days 7 and 28 post mOPV2 challenge by 62.0%, 64.9% and 69.1% in SP-2, GSK-2 and BBio-2 groups, respectively (Fig. 2). The SIE was 2.3 (95% CI: 2.1–2.6) for SP-2, 2.2 (95% CI: 1.7–2.5) for GSK-2 and 1.8 (95% CI: 1.5–2.3) for BBio-2 (p < 0.01), further indicating equivalence in the composite shedding measure between all manufacturers.

### 3.5 Safety

There were no fatalities over the entire study period. Of 73 SAEs reported in 50 subjects in the 6 groups, none were considered to be related to the study vaccines. Most SAEs (69 of 73, 95%) were Infections and Infestations (MEDRA code 10021881). Of 134 IMEs reported in 100 subjects, 107 (80%) were General Disorders and Administration Site Conditions (MEDRA code 10018065). No severe solicited or unsolicited AEs were reported.

## 4 Discussion

This report is the first to offer evidence on the comparability of humoral and intestinal immune responses, and safety profiles of three globally available WHO-prequalified IPV vaccines. Each of these vaccines induces high seroconversion rates against polioviruses in children previously immunized with 3 doses of bOPV. Overall type 2 seroconversion, a measure of IPV performance, occurred in >79% of infants after one dose and in 99.8% after two doses. In infants seronegative before IPV at 14 weeks of age, type 2 seroconversion was attained in 92.7% four weeks later, persisting to 82.7% 22 weeks later in a parallel group.

Previous studies comparing IPV used for primary infant immunization as a standalone or combination vaccine component have demonstrated >90% seropositivity after 2 doses [10], [11], [12], [13], [14], implying comparability [15]. However, our head-to-head comparison of three standalone IPVs used in conjunction with bOPV - important in view of the recent global switch from tOPV to bOPV- was designed to test their equivalence on humoral and intestinal immunity. While in many instances the three IPVs reached equivalence, this was not uniform for all the endpoints. The lack of conclusive evidence for equivalence between some of the BBio humoral responses, especially after one dose, may have been influenced by the higher rates of maternal antibody observed at baseline even when the level of maternal antibody was not significantly different between groups (Supplementary data).

We previously showed one dose of SP IPV has a small but significant decrease in shedding of poliovirus 2 compared with controls given bOPV only [6], [7]. Doses of SP and GSK IPVs showed similar decreases in type 2 viral shedding following mOPV2 challenge, an effect that was most pronounced after the second dose as previously reported [6]. The BBio group showed a slightly greater decrease in the proportion of infants shedding after mOPV2 challenge compared with the SP and GSK groups. Typically, highest viral shedding is observed 7–10 days post oral challenge, a period considered important for impact on person-to-person transmission. It is unclear whether the smaller proportion of subjects excreting type 2 vaccine virus observed in the BBio IPV three weeks post-challenge translates into any clinically and epidemiologically significant advantage in the community transmission of polioviruses upon exposure. Nevertheless, when considering the shedding index endpoint (SIE), a composite measure of the viral load and the time of excretion, all IPVs were equivalent in their impact on excretion, within a margin of 1.0 log_10_.

As of November 7, 2016, 105 of 126 (83%) countries using only OPV at the beginning of 2013 had introduced IPV, resulting in 173 of 194 (89%) WHO member states using IPV, primarily due to supply shortages [16]. Therefore, our findings that all three IPV vaccines achieve high immunogenicity and safety standards should reassure policy and decision-makers making choices for IPV for national and regional immunization schedules.

We must acknowledge some important limitations. This study did not assess the interchangeability of different IPVs in the same infants. The sample size was powered for the primary objectives, and while allowing us to test the equivalence between IPVs, it limited our ability to demonstrate manufacturer equivalence for all endpoints. Finally, we used the different IPVs as standalone vaccines, but many infants, especially in middle- and high-income countries, typically receive their IPV in combinations with diphtheria-tetanus-pertussis, variously including hepatitis B and *Haemophilus influenzae* type b components. Such vaccines are approved for licensure on the basis that the combination does not significantly impact the immunogenicity of the individual components, so it is unlikely that IPV administration in this form would generate results different from those we have observed.

## 5 Conclusions

Although not equivalent for all endpoints evaluated, possibly due to the limited power to make these comparisons, each IPV produced similarly reassuring immunogenicity and safety results. Most countries now provide at least one IPV dose in sequential or mixed bOPV-IPV schedules, and current WHO-prequalified IPV vaccines should be considered comparable. For poliovirus type 2, one IPV dose at 14 weeks of age induced seroconversion in 80% of infants; most who remained seronegative were ready to respond upon type 2 exposure to become protected against paralytic disease. Two doses of IPV protected virtually all infants. Minor differences in seroconversion and viral shedding rates observed between manufacturers are unlikely to be clinically or epidemiologically relevant for achieving and sustaining global polio eradication.

## Conflicts of interest

This study was funded by the Bill & Melinda Gates Foundation (BMGF). ASB is a full-time employee at BMGF and contributed to the study design, data interpretation and writing of the manuscript. Other authors confirm they have no conflicts of interest to declare.
