# Supplementary material for: Inactivated polio vaccines from three different manufacturers have equivalent safety and immunogenicity when given as 1 or 2 additional doses after bivalent OPV: Results from a randomized controlled trial in Latin America
Source: Vaccine. 2017 Jun 16;35(28):3591–7. doi: 10.1016/j.vaccine.2017.04.041 (PMC5464088; doi:10.1016/j.vaccine.2017.04.041)
Supplement: Supplementary data 1 [file mmc1.docx]

| **Supplementary table 1.** Components of the IPV vaccines administered in the six study groups. | | | | | |  |
| --- | --- | --- | --- | --- | --- | --- |
| **Manufacturer** | **Vaccine** | **Dose** | **Serotype 1** | **Serotype 2** | **Serotype 3** | **Other components** |
| **Sanofi Pasteur** | SP IPV | 0.5 mL | 40 D antigen units of Mahoney strain | 8 D antigen units of MEF-1 strain | 32 D antigen units of Saukett strain | 0.5% 2-phenoxyethanol  0.02% of formaldehyde  5 ng of neomycin  200 ng of streptomycin  25 ng of polymixin B |
| **GlaxoSmithKline** | GSK IPV | 0.5 mL | 40 D antigen units of Mahoney strain | 8 D antigen units of MEF-1 strain | 32 D antigen units of Saukett strain | 0.5 mg 2-phenoxyethanol  0.01 mg of formaldehyde  neomycin, polymixin B, polysorbate 80  medium 199 |
| **Bilthoven Biologicals** | BBio IPV | 0.5 mL | 40 D antigen units of Mahoney strain | 8 D antigen units of MEF-1 strain | 32 D antigen units of Saukett strain | 2.5 mg 2-phenoxyethanol  12.5 mcg of formaldehyde traces of phosphate buffer and medium 199 |
|  |  |  |  |  |  |  |
