# Supplementary material for: Inactivated polio vaccines from three different manufacturers have equivalent safety and immunogenicity when given as 1 or 2 additional doses after bivalent OPV: Results from a randomized controlled trial in Latin America
Source: Vaccine. 2017 Jun 16;35(28):3591–7. doi: 10.1016/j.vaccine.2017.04.041 (PMC5464088; doi:10.1016/j.vaccine.2017.04.041)
Supplement: Supplementary data 2 [file mmc2.docx]

| **Supplementary table 2.** Median log neutralizing antibody titers (95% CI) against the three polio serotypes in the six study groups given 1 or 2 doses of IPV from the three different manufacturers (PP data-set), with bOPV at weeks 6, 10 and 14 weeks | | | | | | | | | | | |
| --- | --- | --- | --- | --- | --- | --- | --- | --- | --- | --- | --- |
| **One dose of IPV at week 14** (mOPV2 at week 18) | | | | | | **Two doses of IPV at weeks 14 and 36** | | | | | |
| Week | | **SP-1** | **GSK-1** | **BBio-1** | **Combined** | Week | **SP-2** | **GSK-2** | **BBio-2** | | **Combined** |
|  | **Serotype 1** | | | | | | | | | | |
| **6** | | *196*  **2.8**  (2.5–3.5) | *46*  **3.0**  (2.5–3.5) | *46*  **3.5**  (2.8–4.2) | *288*  **3.0**  (2.8–3.5) | **6** | *192*  **3.2**  (2.5–3.5) | *174*  **3.2**  (2.8–3.5) | *169*  **3.2**  (2.8–3.5) | | *535*  **3.2**  (2.8–3.5) |
| **14** | | *194*  **> 10.5**  (10.5–10.5) | *46*  **> 10.5**  (10.5–10.5) | *46*  **> 10.5**  (10.5–10.5) | *286*  **> 10.5**  (10.5–10.5) | **14** | *192*  **> 10.5**  (10.5–10.5) | *174*  **> 10.5**  (10.5–10.5) | *169*  **> 10.5**  (10.5–10.5) | | *535*  **> 10.5**  (10.5–10.5) |
| **18** | | *194*  **> 10.5**  (98.6–100) | *46*  **> 10.5**  (94.5–100) | *45*  **> 10.5**  (94.3–100) | *285*  **> 10.5**  (10.5–10.5) | **36** | *192*  **> 10.5**  (10.5–10.5) | *173*  **> 10.5**  (10.5–10.5) | *169*  **> 10.5**  (10.5–10.5) | | *534*  **> 10.5**  (10.5–10.5) |
| **19** | | *193*  **> 10.5**  (10.5–10.5) | *45*  **> 10.5**  (10.5–10.5) | *45*  **> 10.5**  (10.5–10.5) | *283*  **> 10.5**  (10.5–10.5) | **40** | *192*  **> 10.5**  (10.5–10.5) | *174*  **> 10.5**  (10.5–10.5) | | *169*  **> 10.5**  (10.5–10.5) | *535*  **> 10.5**  (10.5–10.5) |
|  | **Serotype 2** | | | | | | | | | | |
| **6** | | *196*  **3.3**  (2.8–3.5) | *46*  **3.2**  (2.5–4.08) | *46*  **3.5**  (2.8–4.5) | *288*  **3.5**  (3.2–3.5) | **6** | *192*  **3.2**  (2.8–3.5) | *174*  **3.5**  (3.2–3.5) | *169*  **3.2**  (2.8–3.5) | | *535*  **3.2**  (3.2–3.5) |
| **14** | | *194*  **< 2.5**  (2.5–2.5) | *46*  **< 2.5**  (2.5–2.8) | *46*  **2.7**  (2.5–3.2) | *286*  **< 2.5**  (2.5–2.5) | **14** | *192*  **< 2.5**  (2.5–2.5) | *174*  **< 2.5**  (2.5–2.5) | *169*  **< 2.5**  (2.5–2.5) | | *535*  **< 2.5**  (2.5–2.5) |
| **18** | | *194*  **4.8**  (4.5–5.2) | *46*  **5.2**  (4.3–6.2) | *45*  **4.8**  (4.2–5.5) | *285*  **4.8**  (4.5–5.2) | **36** | *192*  **4.0**  (3.8–4.5) | *173*  **4.2**  (3.5–4.5) | *169*  **4.5**  (4.2–5.2) | | *534*  **4.2**  (3.8–4.5) |
| **19** | | *193*  **7.5**  (6.8–8.2) | *45*  **6.8**  (6.2–7.8) | *45*  **8.5**  (7.2–9.2) | *283*  **7.5**  (6.8–7.8) | **40** | *192*  **> 10.5**  (10.5–10.5) | *174*  **> 10.5**  (10.5–10.5) | | *169*  **> 10.5**  (10.5–10.5) | *535*  **> 10.5**  (10.5–10.5) |
|  | **Serotype 3** | | | | | | | | | | |
| **6** | | *196*  **< 2.5**  (2.5–2.5) | *46*  **< 2.5**  (2.5–2.5) | *46*  **< 2.5**  (2.5–2.5) | *288*  **< 2.5**  (2.5–2.5) | **6** | *192*  **< 2.5**  (16.0–25.5) | *174*  **< 2.5**  (20.8–31.7) | | *169*  **< 2.5**  (19.3–30.1) | *535*  **< 2.5**  (20.5–26.5) |
| **14** | | *194*  **> 10.5**  (10.5–10.5) | *46*  **> 10.5**  (10.5–10.5) | *46*  **> 10.5**  (10.5–10.5) | *286*  **> 10.5**  (10.5–10.5) | **14** | *192*  **> 10.5**  (10.5–10.5) | *174*  **> 10.5**  (10.5–10.5) | *169*  **> 10.5**  (10.5–10.5) | | *535*  **> 10.5**  (10.5–10.5) |
| **18** | | *194*  **> 10.5**  (10.5–10.5) | *46*  **> 10.5**  (10.5–10.5) | *45*  **> 10.5**  (10.5–10.5) | *285*  **> 10.5**  (10.5–10.5) | **36** | *192*  **> 10.5**  (10.5–10.5) | *173*  **> 10.5**  (10.5–10.5) | *169*  **> 10.5**  (10.5–10.5) | | *534*  **> 10.5**  (10.5–10.5) |
| **19** | | *193*  **> 10.5**  (10.5–10.5) | *45*  **> 10.5**  (10.5–10.5) | *45*  **> 10.5**  (10.5–10.5) | *283*  **> 10.5**  (10.5–10.5) | **40** | *192*  **> 10.5**  (10.5–10.5) | *174*  **> 10.5**  (10.5–10.5) | *169*  **> 10.5**  (10.5–10.5) | | *535*  **> 10.5**  (10.5–10.5) |
